# Short‐term control of diet affects cisplatin‐induced acute kidney injury through modulation of mitochondrial dynamics and mitochondrial GSH

**DOI:** 10.14814/phy2.15348

**Published:** 2022-06-24

**Authors:** Ji Su Kim, Yong Kwon Han, Min Jung Kong, Kwon Moo Park

**Affiliations:** ^1^ Department of Anatomy and BK21 Plus School of Medicine, Kyungpook National University Daegu Republic of Korea; ^2^ Cardiovascular Research Institute, Kyungpook National University Daegu Republic of Korea

**Keywords:** acute kidney injury, apoptosis, cisplatin, fasting, high‐fat diet, mitochondrial damage, oxidative stress

## Abstract

Obesity affects acute kidney injury (AKI) induced by various clinical settings, including transplantation and cisplatin‐cancer therapy. However, the effect of short‐term food intake change remains to be defined. Here, we investigated the effects of short‐term high‐fat diet intake and food restriction on cisplatin‐induced AKI. Mice were fed either a high‐fat diet (HFD) or a low‐fat diet (LFD) for 11 days or were not fed for 40 hh (fasting), before cisplatin administration. Cisplatin‐induced functional and structural damages to kidneys in both HFD‐ and LFD‐fed mice, with greater damages in HFD‐fed mice than LFD‐fed mice. HFD decreased mitochondrial total glutathione (tGSH) level, along with increases in the plasma and kidney cholesterol levels. Cisplatin caused the increase of kidney cholesterol levels and oxidative stress, along with the decrease of mitochondrial tGSH levels. In addition, cisplatin‐induced mitochondrial damage and apoptosis of tubular cells in both HFD‐ and LFD‐fed mice. An increase of Fis1 (mitochondria fission 1 protein), whereas a decrease of Opa1 (mitochondria fusion 1 protein) occurred by cisplatin. These cisplatin effects were greater in HFD‐fed mice than in LFD‐fed mice. Administration of mitochondria‐specific antioxidant treatment during HFD feeding inhibited these cisplatin‐induced changes. Fasting for 40 h also significantly reduced the cisplatin‐induced changes mentioned above. These data demonstrate that short‐term HFD intake worsens cisplatin‐induced oxidative stress by the reduction of mitochondrial tGSH, resulting in increased cisplatin‐induced nephrotoxicity. These data newly indicate that the control of calorie intake, even for a short period, affects kidney susceptibility to injury. Although most studies described the effects of a long‐term high‐fat diet on the kidneys, in this study, we found that even if a high‐fat diet was consumed for a short‐term, physiological changes and mitochondria tGSH decrease in the kidneys, and consequently increased cisplatin‐nephrotoxic susceptibility. These data suggest the association of calorie intake with kidney susceptibility to cisplatin.

## Graphical abstract

Our findings indicate that control of calorie intake, even for a short period, could be considered an adjunct to AKI, which is inevitable in clinical settings such as cisplatin‐chemotherapy, transplantation, cardiac bypass surgery, and so on.

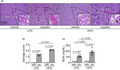

## 1 INTRODUCTION

Cisplatin [cis‐diamminedichloroplatinum (II)] is one of the most effective and widely used anticancer agents. However, its use is limited due to its side effects, which include nephrotoxicity linked with acute kidney injury (AKI), as ~30% of patients experience renal damage after one high dose of cisplatin (Arany & Safirstein, 2003; Pabla & Dong, 2008). Cisplatin metabolites in the body are primarily eliminated through the kidneys (Pabla & Dong, 2008; Peres & da Cunha Jr., 2013). During this elimination, since cisplatin metabolites accumulate in the mitochondria of kidney tubular epithelial cells (Bajwa et al., 2015; Pabla & Dong, 2008; Santos et al., 2008), this accumulated cisplatin causes dysfunction in the electron transport chain I–IV complex, which results in excessive production of reactive oxygen species (ROS) in the mitochondria (Kruidering et al., 1997). Simultaneously, cisplatin metabolites impair mitochondrial antioxidant systems, such as reducing mitochondrial glutathione (GSH) levels (Kong et al., 2018; Santos et al., 2007; Yonezawa, 2012). Impairments in mitochondrial redox balance due to the abnormal production and removal of ROS result in mitochondrial dysfunction, oxidative stress, and consequently, cellular damage (Brady et al., 1990; Cilenti et al., 2005; Kong et al., 2018; Kong et al., 2019; Noh et al., 2017). Thus, antioxidants are often used in combination with cisplatin for chemotherapy to reduce the side effects of cisplatin (Conklin, 2000, 2004; Longchar & Prasad, 2015; Pace et al., 2003; Weijl et al., 2004).

Calorie intake affects the cellular redox status due to changes in cellular metabolism. Studies have demonstrated that long‐term high‐calorie intake influences cellular susceptibility to various stimuli (Ali et al., 2016; Noh et al., 2020; Ribeiro et al., 2017; Sun et al., 2020; Zalewska et al., 2019). Interestingly, long‐term high‐calorie intake increases the susceptibility of the kidneys to cisplatin‐induced damage, whereas food restriction decreases this susceptibility (Gunebakan et al., 2020; Ning et al., 2013; Ribeiro et al., 2017). Accumulating evidence has revealed that cell susceptibility linked to calorie intake is associated with cellular redox balancing systems, including ROS‐producing and/or ROS‐scavenging systems (Noh et al., 2020; Sun et al., 2020; Zalewska et al., 2019). Furthermore, excessive caloric intake can affect tissue mitochondrial dynamics and dysfunction. Lionetti observed the presence of numerous small circular mitochondria in the liver of HFD‐fed rats and reported a decrease in mitochondria fusion‐related proteins (Opa1 and Mfn2) and an increase in mitochondria fission‐related proteins (Fis1 and Drp1) (Lionetti et al., 2014). In addition, Holmstrom et al. reported that mitochondrial dysfunction in the livers of obese diabetic mice was associated with an enhanced mitochondrial fission process (Holmstrom et al., 2012), and Langley et al. observed that Fis1 expression was upregulated and increased lipid peroxidation in the spinal cords of mice fed a high‐fat diet (Langley et al., 2020). Andrich et al. reported that HFD‐feeding for 2 weeks causes the increase of oxidative stress in skeletal muscles of rats (Andrich et al., 2019). However, the effects of short‐term excessive caloric intake on cellular susceptibility to various stimuli are rarely studied.

Therefore, we investigated the effects of short‐term high‐fat diet intake and food restriction on cisplatin‐induced AKI and its‐related mechanisms. In the present study, we found that short‐term high‐fat diet intake increases kidney susceptibility to cisplatin, whereas food restriction decreases that susceptibility, and that these effects are related to the cellular redox system, mitochondrial damage, and apoptosis. In addition, mitochondrial antioxidant treatment during high‐fat diet intake prevents the increase in kidney susceptibility induced by a high‐fat diet. These data indicate the association of calorie intake with kidney susceptibility to cisplatin.

## 2 MATERIALS AND METHODS

### 2.1 Animal experiments

All experiments were performed in 9‐week‐old male C57BL/6 mice (Koatech, Pyeongtaek, Gyeonggi‐do, Korea). The animal study was approved by the Institutional Animal Care and Use Committee of Kyungpook National University. Before the experiments were initiated, the mice were provided free access to water and standard chow (Envigo, Indianapolis, IN, USA). Mice were randomly assigned and fed either a high‐fat diet containing 60% kcal from fat (HFD; TD.06414, Harlan Laboratories, Inc., Madison, WI, USA) or a low‐fat diet containing 10% kcal from fat (LFD; TD.94048, Harlan Laboratories) for 14 days. Some mice were administered 2‐(2,2,6,6‐Tetramethylpiperidin‐1‐oxyl‐4‐ylamino)‐2‐oxoethyl) triphenylphosphonium chloride (Mito‐TEMPO, a mitochondria‐specific antioxidant, 0.7 mg/kg body weight; Sigma‐Aldrich) daily beginning 4 days after HFD or LFD feeding until sacrifice. Cisplatin (*Cis*‐diamminedichloroplatinum II, 20 mg/kg body weight; Sigma‐Aldrich, St. Louis, MO, USA) or vehicle (cisplatin‐dissolving solution as a control) was administered on the 11th day after the change in diet. Some mice, which were provided free access to water and standard chow, were fasted for 40 hours (fasting) and then administered either cisplatin (20 mg/kg BW) or vehicle. After cisplatin injection, the mice were again fed standard chow. Finally, the mice were euthanized with an overdose of pentobarbital sodium (150 mg/kg BW), which was injected 3 days after cisplatin administration. Kidneys were either snap‐frozen in liquid nitrogen for biochemical analysis or perfusion‐fixed in PLP (4% paraformaldehyde, 75 mM L‐lysine, 10 mM sodium periodate; Sigma‐Aldrich) for histological studies.

### 2.2 Blood analysis

Blood was collected from mice using a heparinized syringe. Concentrations of cholesterol, high‐density lipoprotein (HDL), triglyceride (TG), and blood urea nitrogen (BUN) in the plasma were determined using a VITROS 250 Chemistry Analyzer (Johnson & Johnson, Rochester, NY, USA) according to the manufacturer's instructions.

### 2.3 Histology analysis

PLP‐fixed kidneys were paraffin‐embedded and cut into 3‐μm‐thick sections using a microtome (Leica, Bensheim, Germany). Kidney sections were stained with periodic acid‐Schiff (PAS) according to the manufacturer's instructions. The sections were observed under a Leica microscope (Leica), and images were captured using i‐Solution software (IMT, Cicero, NY, USA). Kidney damage was scored in a blind manner according to the following criteria as previously described (Kong et al., 2019; Park et al., 2001).

### 2.4 Oil Red O staining

Fixed frozen kidney tissue was cut into 10‐μm‐thick sections, and subjected to Oil Red O (Sigma‐Aldrich) staining following the manufacturer's protocol. Briefly, sections were rinsed in distilled water for 5 min, 60% isopropanol for 5 min, and then subjected to Oil Red O solution for 10 min, and then rinsed in 60% isopropanol and distilled water for 5 min each. This section was counterstained with hematoxylin for 30 s. Images were captured using a microscope.

### 2.5 Determination of tissue cholesterol

First, the kidney was homogenized in PBS and then this homogenate was mixed with chloroform and methanol as the ratio of tissue lipid was extracted by chloroform:methanol:PBS extraction (4:2:1). This mixture was centrifuged at 4200 *g* (Thermo Fisher Scientific, Waltham, MA, USA) at 4°C for 10 min, and then the bottom phase of the centrifugate was transferred into a new tube and evaporated for dryness. The dried lipid residue was suspended in 1% Triton‐X 100 in absolute ethanol. Tissue cholesterol levels were determined using a commercially available kit (Cell Biolabs Inc., San Diego, CA, USA) following the manufacturer's protocol.

### 2.6 Western blot analysis

Western blot analysis were performed as described previously (Park et al., 2001; Park et al., 2002). Antibodies were anti‐4‐hydroxynonenal (4‐HNE; ab48506, Abcam, Cambridge, MA, USA), anti‐manganese superoxide dismutase (MnSOD; 574,516, Calbiochem, San Diego, CA, USA), anti‐isocitrate dehydrogenase 2 (IDH2; sc‐134,923, Santa Cruz Biotechnology, Santa Cruz, CA, USA), anti‐fission 1 (Fis1; SAB1405076, Sigma‐Aldrich), anti‐optic atrophy 1 (Opa1; 612,607, BD Bioscience, San Diego, CA, USA), cytochrome c oxidase subunit IV (COX IV; ab33985, Abcam), and anti‐glyceraldehyde 3‐phosphate dehydrogenase (GAPDH; NB600‐502, NOVUS, Littleton, CO, USA).

### 2.7 Measurement of hydrogen peroxide

H_2_O_2_ levels in kidney tissues were determined using the ferric oxide‐sensitive dye, xylenol orange (Sigma‐Aldrich), as previously described (Noh et al., 2020). H_2_O_2_ oxidizes iron (II) to iron (III) in the presence of sorbitol, which acts as a catalyst. Iron (III) forms a purple complex with xylenol orange. Absorbance was measured at a wavelength of 560 nm.

### 2.8 Mitochondria isolation from kidney tissue

Mitochondrial and cytosolic fractions were prepared as described previously (Frezza et al., 2007). Mitochondrial fraction was confirmed by Western blot analysis using COX‐IV antibody.

### 2.9 Measurement of total glutathione (tGSH) and oxidized glutathione (GSSG) levels in the kidney mitochondria

Total GSH (tGSH) and GSSG levels were measured as described previously (Akerboom & Sies, 1981). Measurement of tGSH and GSSG is based on the enzymatic recycling method for quantification of glutathione; GR reduces oxidized glutathione (GSSG) to reduced glutathione (GSH). The sulfhydryl group of GSH reacts with DTNB (5,5′‐dithiobis‐2‐nitrobenzoic acid) to produce a yellow‐colored 5‐thio‐2‐nitrobenzoic acid (TNB) that absorbs at 405 nm. The rate of TNB production is directly proportional to the concentration of glutathione in the sample. The ratio of GSSG to tGSH was measured using the glutathione (GSSG/GSH) detection kit (Enzo Life Sciences, Farmingdale, New York, USA) according to the manufacturer's instructions. In brief, freshly isolated mitochondrial fractions were suspended in ice‐cold 5% metaphosphoric acid (20 μl/mg tissue) and centrifuged at 12,000 × g for 15 min at 4 °C. Supernatants reacted to the freshly prepared reaction mix. The absorbances were detected at 414 nm every minute for 10 min. Determination of GSSG was the same protocol with GSH assay with an exception in which mitochondrial fraction was suspended in 5% metaphosphoric acid containing 2 M 4‐vinyl pyridine.

### 2.10 Transmission electron microscopy

Kidneys were perfusion‐fixed with 2.5% glutaraldehyde via the abdominal aorta, then stored overnight in fixative at 4°C. Samples were cut into 1‐mm pieces, washed 3 times in 0.1 M phosphate buffer, and then postfixed in aqueous 2% osmium tetroxide for 90 min. After three washes in 0.1 M phosphate buffer, the samples were dehydrated through a graded series of 50%–100% ethanol and 100% propylene oxide and then infiltrated in 1:1, 1:2, and 1:3 mixtures of propylene oxide: Epon Resin 828 (Polysciences Inc., Warrington, PA, USA) for 1 h. After samples were incubated in 100% Epon Resin 828 for over 8 h, the samples were then embedded in molds and cured at 35°C and 45°C for 12 h, followed by additional hardening at 60°C for 2 days. Ultrathin (60 nm) sections were double‐stained with 2% uranyl acetate and 1% lead citrate. Sections were visualized using a transmission electron microscope (H‐7000; Hitachi, Yokohama, Japan) at 75 kV. Electron micrographs of mitochondria were captured from the proximal tubule cells in the cortex.

### 2.11 Terminal deoxynucleotidyl transferase dUTP nick end labeling (TUNEL) assay

To detect apoptotic cells, TUNEL assays were performed using an in situ cell death detection kit (Roche Molecular Biochemicals, Indianapolis, IN, USA) according to the manufacturer's instructions. Briefly, kidney sections were incubated with the TUNEL reagent mixture at room temperature for 30 min and washed three times in PBS for 5 min each time. TUNEL‐positive cells were quantified under a microscope (Leica).

### 2.12 Statistical analysis

All data were analyzed using GraphPad Prism 6 software (GraphPad, San Diego, CA, USA). Results are expressed as the mean ± standard deviation. Statistical differences among the groups were assessed using Student's *t*‐test and two‐way ANOVA with repeated measures followed by post hoc Bonferroni's multiple comparisons test. Differences were considered significant at a *p* < 0.05.

## 3 RESULTS

### 3.1 Short‐term HFD intake exacerbates kidney damage by cisplatin

**FIGURE 1:**
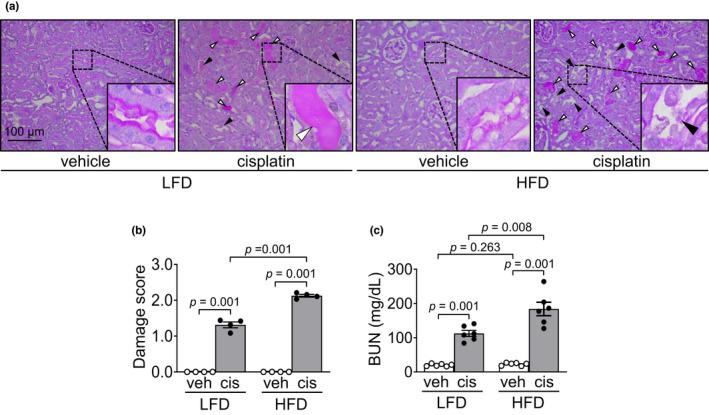
HFD feeding exacerbates cisplatin‐induced kidney damage. Mice were allowed free access to water and standard chow before experiments were initiated. From the start, mice were fed either a high‐fat diet containing 60% fat (HFD) or a low‐fat diet containing 10% fat (LFD) for 14 days. Cisplatin (20 mg/kg body weight) or vehicle (cisplatin‐dissolving solution as a control) was administered to the mice on the 11th day after the change in diet. Kidneys and blood were collected 3 days after those administrations. (a) Kidney sections were subjected to PAS staining. The white arrowhead indicates congested tubules (damage score: 2). The black arrowhead indicates disrupted tubules (damage score: 3). (b) Tubular damage was scored as described in the Materials and Methods. (c) Concentrations of blood urea nitrogen (BUN) in the plasma was determined 3 days after those administrations. Results are expressed as the means ± SEM (*n* = 4–6).

To determine the effect of short‐term high‐fat diet (HFD) intake on cisplatin‐induced AKI, we evaluated kidney damage 3 days after cisplatin administration. Cisplatin caused brush border loss and disruption of kidney tubular epithelial cells, as well as collapse, dilation, and congestion of kidney tubules in both HFD‐ and LFD‐fed mice (Figure 1a). The extent of kidney damage was greater in HFD‐fed than LFD‐fed mice (Figure 1a,b). Concomitant with the extent of histological damage, blood urea nitrogen (BUN) as increased in both HFD‐fed and LFD‐fed mice after cisplatin injection, and these increases were greater in HFD‐fed mice than in LFD‐fed mice (Figure 1c). No significant differences were observed in morphology or the BUN level between HFD‐fed and LFD‐fed mice after vehicle injection (Figure 1a–c). These data indicate that short‐term HFD feeding increases kidney susceptibility to cisplatin.

### 3.2 **Short‐term** **HFD** **intake increases lipid levels** in kidney tissue

**FIGURE 2:**
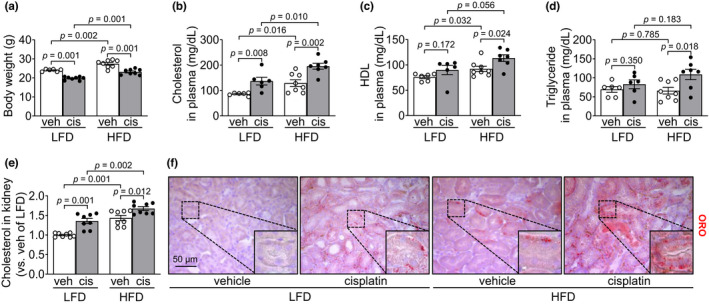
Effect of HFD on cholesterol and triglyceride in the blood, and lipid accumulation in the kidney. Mice were allowed free access to water and standard chow before experiments were initiated. From the start, mice were fed either a high‐fat diet containing 60% fat (HFD) or a low‐fat diet containing 10% fat (LFD) for 14 days. Cisplatin (20 mg/kg body weight) or vehicle (cisplatin‐dissolving solution as a control) was administered to the mice on the 11th day after the change in diet. Kidneys and blood were collected 3 days after those administrations. (a) Body weights were measured 3 days after cisplatin or vehicle administration. (b–d) concentrations of cholesterol, high‐density lipoprotein (HDL), and triglyceride were determined 3 days after those administrations. (e) Kidney cholesterol levels were determined as described in the Mterials and Methods. (f) Kidney sections were subjected to Oil Red O (ORO) staining and images were captured in the cortex. Results are expressed as the means ± SEM (*n* = 4–8).

First, we investigated body weight (BW) and blood chemistry 3 days after either cisplatin or vehicle administration, that is, 14 days after either HFD‐ or LFD‐feeding. HFD‐ and LFD‐fed mice were fed approximately 11.2 kcal and 8.8 kcal per day, respectively. Body weight and concentrations of total cholesterol and HDL, but not a triglyceride, were significantly higher in HFD‐fed mice than in LFD‐fed mice (Figure 2a–d). Cisplatin injection decreased BW in both HFD‐fed and LFD‐fed mice (Figure 2a). Cisplatin injection increased the plasma cholesterol concentration in both HFD‐fed and LFD‐fed mice and the plasma HDL and triglyceride concentration in HFD‐fed mice (Figure 2b–d).

Next, we determined the cholesterol levels in the kidneys. Kidney cholesterol levels in HFD‐fed mice were higher than those in LFD‐fed mice (*p* = 0.001) (Figure 2e). Cisplatin injection increased cholesterol levels in both HFD‐fed and LFD‐fed mice with a greater increase in the HFD‐fed mice than in the LFD‐fed mice (*p* = 0.002) (Figure 2e). In addition, we determined the lipid accumulation in the kidneys by an Oil Red O (ORO) staining. Consistent with cholesterol accumulation results, The ORO‐positive signal was greater in the kidneys of HFD‐fed mice than of LFD‐fed mice (Figure 2f). Cisplatin administration increased ORO‐positive signals in both HFD‐ and LFD‐fed mice with a higher increase in the HFD‐fed mice than in the LFD‐fed mice (Figure 2f). ORO‐positive signals were mainly observed on the basal part of cytosol in the proximal tubule cells (Figure 2f).

These data indicate that short‐term HFD‐feeding increases blood cholesterol concentration and tissue lipid accumulation, subsequently augmenting cisplatin‐induced increases in the lipid levels of both blood and tissue. This suggests that increased lipid levels due to HFD‐feeding are associated with increased susceptibility of HFD‐fed mice kidneys.

### 3.3 Short‐term HFD intake exacerbates cisplatin‐induced oxidative stress in the kidneys

**FIGURE 3:**
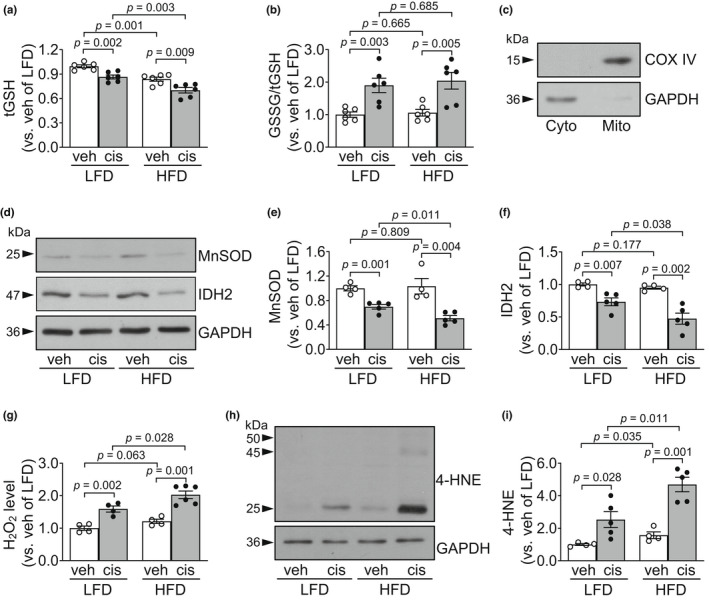
HFD feeding exacerbates cisplatin‐induced kidney oxidative stress. Mice were allowed free access to water and standard chow before experiments were initiated. From the start, mice were fed either a high‐fat diet containing 60% fat (HFD) or a low‐fat diet containing 10% fat (LFD) for 14 days. Cisplatin (20 mg/kg body weight) or vehicle (cisplatin‐dissolving solution as a control) was administered to the mice on the 11th day after the change in diet. Kidneys and blood were collected 3 days after those administrations. (a, b) Total GSH (tGSH) and oxidized GSH (GSSG) amounts were determined in the mitochondrial fractions. (b, c) Fractions were confirmed by western blot analysis using anti‐COX VI antibody for the mitochondria and anti‐GAPDH antibody for the cytosol. (d–f, h, i) MnSOD, IDH2, and 4‐HNE level in the kidney tissues were determined by western blot analysis. GAPDH served as a loading control. (e, f, i) The densities of the bands were measured using ImageJ (g) H_2_O_2_ was measured in the kidney tissues. Results are expressed as the means ± SEM (*n* = 4–6).

The increase of cholesterol in the mitochondria causes the reduction of mitochondrial total glutathione (tGSH, one of the most important antioxidant molecules). To test that increased lipid accumulation in the mitochondrial area of the kidney proximal tubules of HFD‐fed mice affects mitochondrial tGSH levels and oxidative stress, we determined tGSH amount and oxidized glutathione (GSSG) levels in the mitochondrial fractions of kidneys. Mitochondrial tGSH levels in the kidneys of HFD‐fed mice were significantly lower than those in LFD‐fed mice (Figure 3a). Cisplatin decreased tGSH and increased the ratio of GSSG to tGSH in the mitochondrial fraction of kidneys in the HFD‐fed mice and LFD‐fed mice (Figure 3a,b). The reduction of tGSH by cisplatin administration was greater in the HFD‐fed mice than LFD‐fed mice (Figure 3a). Mitochondrial and cytosolic fraction was confirmed through western blot analysis using anti‐cytochrome c oxidase subunit IV (COX IV) for the mitochondria and ‐glyceraldehyde 3‐phosphate dehydrogenase (GAPDH) for the cytosol (Figure 3c).

Next, we measured the expression of mitochondrial antioxidant enzymes in the kidneys: Manganese superoxide dismutase (MnSOD) and isocitrate dehydrogenase 2 (IDH2). The expression levels of MnSOD and IDH2 after cisplatin injection were significantly decreased in the kidneys of both HFD‐fed and LFD‐fed mice compared with the levels after vehicle injection (Figure 3d–f). These decreases in HFD‐fed mice were greater than those in LFD‐fed mice (Figure 3d–f). However, no significant differences were observed in the expression of MnSOD and IDH2 between HFD‐fed mice and LFD‐fed mice after vehicle injection (Figure 3d–f).

Finally, to assess whether increased cisplatin‐induced kidney damage is associated with oxidative stress, we investigated the redox status of the kidney by measuring the H_2_O_2_ and 4‐hydroxynonenal (4‐HNE, an index of lipid peroxidation) level in the kidneys. Cisplatin significantly increased the H_2_O_2_ level in the kidneys in both HFD‐fed and LFD‐fed mice (Figure 3g). These cisplatin‐induced increases were greater in the HFD‐fed mice than in LFD‐fed mice (Figure 3g). Consistent with the H_2_O_2_ increase, 4‐HNE level was also increased in the kidney after cisplatin injection (Figure 3h,i). This increase was greater in the HFD‐fed mice than in LFD‐fed mice (Figure 3h,i). After vehicle administration, the H_2_O_2_ and 4‐HNE levels in HFD‐fed mice were slightly higher compared with those in LFD‐fed mice (*p* = 0.063 in H_2_O_2_ level; *p* = 0.035 in a 4‐HNE level) (Figure 3g–i). These data indicate that an HFD augments cisplatin‐induced oxidative stress by decreasing both mitochondrial tGSH level and mitochondrial antioxidant enzymes.

### 3.4 Short‐term HFD intake exacerbates cisplatin‐induced apoptosis of kidney tubular cells

**FIGURE 4:**
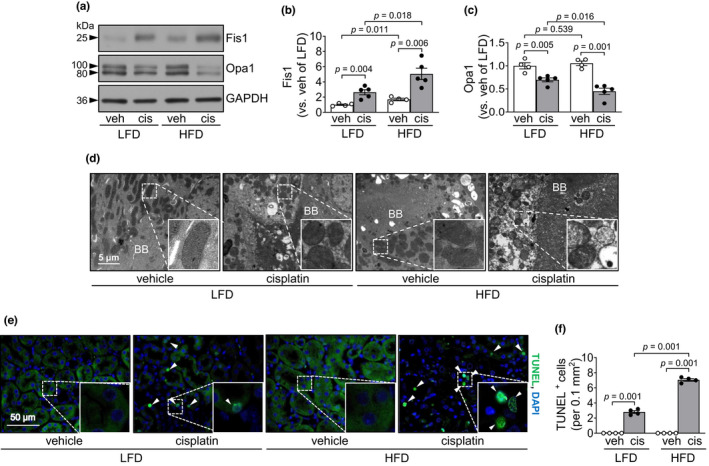
HFD feeding exacerbates cisplatin‐induced tubular cell apoptosis and mitochondrial damage. Mice were allowed free access to water and standard chow before experiments were initiated. From the start, mice were fed either a high‐fat diet containing 60% fat (HFD) or a low‐fat diet containing 10% fat (LFD) for 14 days. Cisplatin (20 mg/kg body weight) or vehicle (cisplatin‐dissolving solution as a control) was administered to the mice on the 11th day after the change in diet. Kidneys and blood were collected 3 days after those administrations. (a) Fis1 and Opa1 expression in the kidneys were analyzed by western blot. GAPDH served as a loading control. (b, c) The densities of the bands were measured using ImageJ. (d) Mitochondrial structure in proximal tubule cells was evaluated by transmission electron microscopy (TEM). BB is a brush borders (BB) of proximal tubule. (e) Apoptosis of kidney tubular cells was evaluated using a terminal deoxynucleotidyl transferase dUTP nick‐end labeling (TUNEL) assay. The white arrowhead indicates a TUNEL‐positive cell (green). DAPI (blue) was used to stain the nuclei. (f) The number of TUNEL‐positive cells was counted under a fluorescence microscope. Results are expressed as the means ± SEM (*n* = 3–5).

Since redox status influences mitochondrial dysfunction and apoptosis (Page et al., 2010; Putti et al., 2015; Sun et al., 2020), we investigated mitochondrial morphology and the expression of fission 1 (Fis1, mitochondrial fission 1 protein) and optic atrophy 1 (Opa1, mitochondrial fusion protein). After cisplatin administration, Fis1 expression significantly increased in the kidney, whereas Opa1 expression decreased (Figure 4a–c). These cisplatin‐induced changes were greater in the kidneys of HFD‐fed than those of LFD‐fed mice (Figure 4a–c). After vehicle administration, Fis1 expression, but not Opa1 expression, was significantly higher in HFD‐fed mice than in LFD‐fed mice (Figure 4a–c).

Next, we determined mitochondrial damage in proximal tubule cells by transmission electron microscopy (TEM). TEM data showed mitochondrial damage accompanied by loss of cristae and disruption of the mitochondrial membrane in both HFD‐ and LFD‐fed mice after cisplatin injection (Figure 4d). This mitochondrial damage was more severe in the HFD‐fed mice than in the LFD‐fed mice (Figure 4d). The length of the mitochondria in the vehicle‐administered HFD‐fed mice was shorter compared with that of the LFD‐fed mice (Figure 4d).

Finally, we determined the level of apoptosis after cisplatin administration. Cisplatin significantly increased TUNEL positivity in kidney tubular cells, and the number of TUNEL‐positive cells was higher in HFD‐fed mice than in LFD‐fed mice (Figure 4e,f).

### 3.5 Mito‐TEMPO treatment during HFD feeding prevents the HFD‐induced increase in kidney damage after cisplatin administration

**FIGURE 5:**
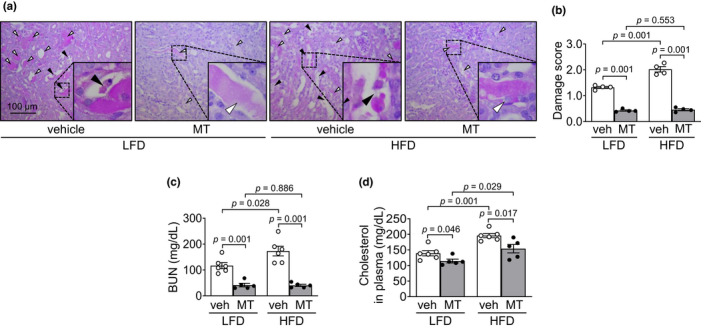
Mitochondrial antioxidant treatment blocks HFD‐induced exacerbation of kidney damage caused by cisplatin. Mice were allowed free access to water and standard chow before experiments were initiated. From the start, mice were fed either a high‐fat diet containing 60% fat (HFD) or a low‐fat diet containing 10% fat (LFD) for 14 days. Some mice were administered either Mito‐TEMPO (MT), a mitochondria‐specific antioxidant, or vehicle every day beginning 4 days after HFD or LFD feeding until sacrifice. Those mice were administered either cisplatin (20 mg/kg BW, i.p.) or vehicle. Kidneys and blood were harvested 3 days after cisplatin injection. (a) Kidney sections were subjected to PAS staining. (b) Tubular damage was scored as described in the Materials and Methods. The white arrowhead indicates a congested tubule (damage score: 2). The black arrowhead indicates a disrupted tubule (damage score: 3). (c, d) Concentrations of blood urea nitrogen (BUN) and cholesterol in the plasma were determined 3 days after cisplatin injection. Results are expressed as the means ± SEM (*n* = 4–6).

To test whether increased susceptibility of HFD‐fed mouse kidneys to cisplatin is associated with increased mitochondrial oxidative stress, mice were administered Mito‐TEMPO (a mitochondria‐specific antioxidant reagent) during the period of HFD‐feeding. Mito‐TEMPO treatment significantly reduced cisplatin‐induced kidney histological and functional damages in both HFD‐ and LFD‐fed mice (Figure 5a–c). These reductions were more prominent in the HFD‐fed mice than in LFD‐fed mice (78.4% in HFD‐fed mice and 67.2% in LFD‐fed mice for tubular damage score; 76.6% in HFD‐fed mice and 64.9% in LFD‐fed mice for BUN; 63.8% in HFD‐fed mice) (Figure 5b,c). Cholesterol tended to decrease after Mito‐TEMPO administration in LFD‐mice, but significantly greater increases were seen in HFD‐fed mice than in LFD‐fed mice (Figure 5d).

**FIGURE 6:**
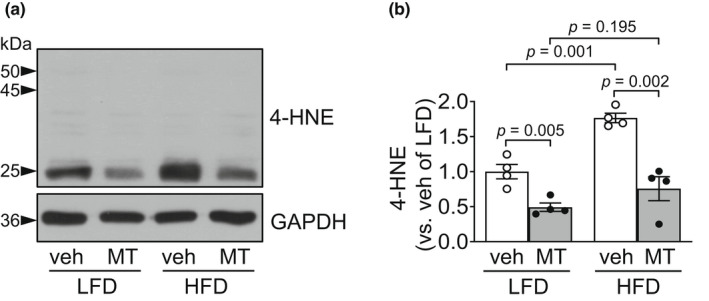
Mitochondrial antioxidant treatment blocks HFD‐induced exacerbation of oxidative stress caused by cisplatin. Mice were allowed free access to water and standard mouse chow before experiments were initiated. From the start, mice were fed either a high‐fat diet containing 60% fat (HFD) or a low‐fat diet containing 10% fat (LFD) for 14 days. Some mice were administered either Mito‐TEMPO (MT), a mitochondria‐specific antioxidant, or vehicle every day beginning 4 days after HFD or LFD feeding until sacrifice. Those mice were administered either cisplatin (20 mg/kg BW, i.p.) or vehicle. Kidneys were harvested 3 days after cisplatin injection. (a) 4‐HNE level was determined in the kidney tissues by western blot analysis. GAPDH served as a loading control. (b) The densities of the bands were measured using ImageJ. Results are expressed as the means ± SEM (*n* = 4).

Consistent with the extent of kidney damage, Mito‐TEMPO administration significantly reduced cisplatin‐induced increases in 4‐HNE level in both HFD‐fed mice and LFD‐fed mice (Figure 6a,b). This reduction was greater in the HFD‐fed mice than in LFD‐fed mice (57.2% in HFD‐fed mice and 50.8% in LFD‐fed mice) (Figure 6a,b).

**FIGURE 7:**
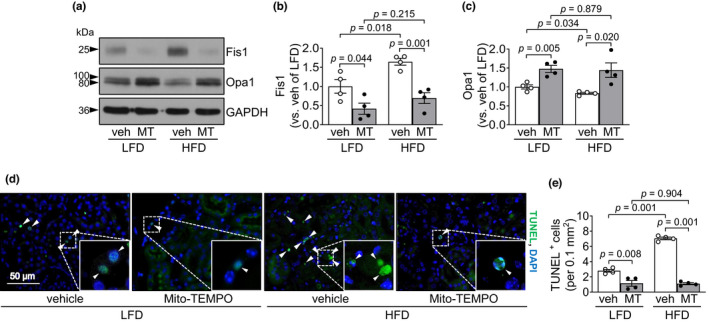
Mitochondrial antioxidant treatment blocks HFD‐induced exacerbation of apoptosis caused by cisplatin. Mice were allowed free access to water and standard chow before experiments were initiated. From the start, mice were fed either a high‐fat diet containing 60% fat (HFD) or a low‐fat diet containing 10% fat (LFD) for 14 days. Some mice were administered either Mito‐TEMPO (MT), a mitochondria‐specific antioxidant, or vehicle every day beginning 4 days after HFD or LFD feeding until sacrifice. Those mice were administered either cisplatin (20 mg/kg BW, i.p.) or vehicle. Kidneys were harvested 3 days after cisplatin injection. (a) Fis1 and Opa1 expression in the kidneys were analyzed by western blot. GAPDH served as a loading control. (b, c) The densities of the bands were measured using ImageJ. (d) Apoptosis of kidney tubule cells was evaluated using a terminal deoxynucleotidyl transferase dUTP nick‐end labeling (TUNEL) assay. The white arrowhead indicates TUNEL‐positive cells (green). DAPI (blue) was used to stain the nuclei. (e) The number of TUNEL‐positive cells was counted under a fluorescence microscope. Results are expressed as the means ± SEM (*n* = 4).

In addition, Mito‐TEMPO treatment prevented the increase in Fis1 and decrease in Opa1 after cisplatin administration (Figure 7a–c). The preventive effects of Mito‐TEMPO were greater in HFD‐fed mice than in LFD‐fed mice (58.2% in LFD‐fed mice and 57.6% in HFD‐fed mice for Fis1; 47.6% in LFD‐fed mice and 73.2% in HFD‐fed mice for Opa1) (Figure 7a–c). Moreover, Mito‐TEMPO significantly prevented cisplatin‐induced increases in the number of TUNEL‐positive cells in both HFD‐fed and LFD‐fed mice, with greater prevention of apoptosis in HFD‐fed mice than in LFD‐fed mice (58.9% in LFD‐fed mice and 84.4% in HFD‐fed mice) (Figure 7d,e). These data indicate that the HFD‐induced increase in kidney damage is mediated by increased mitochondrial oxidative stress.

### 3.6 Food restriction reduces cisplatin‐induced kidney damage

**FIGURE 8:**
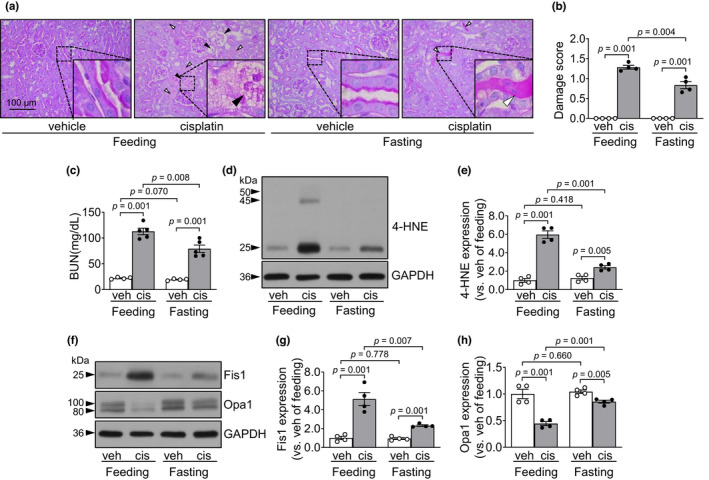
Fasting protects the kidneys against cisplatin. Mice that were fed standard chow were fasted for 40 hours and then administered either cisplatin (20 mg/kg BW) or vehicle. After those administrations, the mice were again fed standard chow. Kidneys and blood were harvested 3 days after cisplatin injection. (a) Kidney sections were subjected to PAS staining. White and black arrowheads indicate congested and disrupted tubules, respectively. (b) Tubular damage was scored as described in the Materials and Methods. The white arrowhead indicates congested tubules (damage score: 2). The black arrowhead indicates disrupted tubules (damage score: 3). (c) Concentrations of blood urea nitrogen (BUN) in the plasma were determined 3 days after those administrations. (d–h) 4‐HNE level, Fis1 and Opa1 expression were determined in the kidney tissues by western blot analysis. GAPDH served as a loading control. (d–h) The densities of the bands were measured using ImageJ. Results are expressed as the means ± SEM (*n* = 4–5).

Lastly, we investigated whether temporal food intake restriction protects cisplatin‐induced kidney damage. For fasting, access to standard chow was blocked for 40 h, and then, mice were administered either cisplatin or vehicle and then again fed with standard mouse chow. Cisplatin increased histological damage of kidney tubular epithelial cells and concentrations of BUN in both fasting and non‐fasting mice, and the kidney damage was much less in fasting than in non‐fasting mice (Figure 8a–c). The level of 4‐HNE was increased in the kidney tissues of both fasting and non‐fasting mice after cisplatin administration, and these increases were less in fasting than in non‐fasting mice (Figure 8d,e). In addition, cisplatin increased Fis1 expression but decreased Opa1 expression (Figure 8f–h). These cisplatin‐induced changes were much less in fasting than in non‐fasting mice (Figure 8f–h). These results indicate that food‐intake restriction ameliorated cisplatin‐induced kidney injury.

## 4 DISCUSSION

Cisplatin is widely used as an anticancer drug, but its use is limited due to side effects, including nephrotoxicity. Therefore, it is necessary to consider how to treat and minimize the side effects. Our present studies have demonstrated that short‐term, for 2 weeks, HFD feeding aggravates cisplatin‐induced acute kidney injury via increases of oxidative stress and mitochondrial damage of kidney tubule cells and that mitochondrial antioxidant treatment during HFD feeding prevented the HFD feeding‐induced increases of cisplatin‐induced AKI. In addition, temporal, for 40 hours, fasting protects the kidney against cisplatin. Although much evidence has demonstrated that calorie intake affects cellular stress including acute kidney injury, most of those evidence has been generated by long‐term effects of calorie intake rather than the short‐term. However, interestingly, in present studies we found that short‐term food intake also affects kidney susceptibility to cisplatin, suggesting that food intake control, even short‐term, could be considered as a tool for the reduction of cisplatin‐induced AKI.

One of the major causes of increased susceptibility due to increased calorie intake, that is excessive energy supply, is oxidative stress due to the impairment of cellular redox balance due to excessive ROS production and/or defect of ROS scavenging systems (Prem & Kurian, 2021; Yu et al., 2008). In contrast, it has been reported that food restriction reduces ROS production and activates the antioxidant system (Hyun et al., 2006; Lettieri‐Barbato et al., 2020; McKiernan et al., 2007; Verweij et al., 2011). In a previous study, we also found that long‐term HFD intake for 12 weeks impairs the function of isocitrate dehydrogenase 2 (IDH2, a mitochondrial NADPH producing enzyme) which plays a mitochondrial antioxidant enzyme in the kidney, resulting in increases in mitochondrial oxidative stress induced by ischemia followed by reperfusion in kidneys (Noh et al., 2020). Prem et al. demonstrated that long‐term HFD intake for 16 weeks deteriorates mitochondrial function, resulting in exacerbation of oxidative stress in renal tissue from ischemia followed by reperfusion (Prem & Kurian, 2021). In the present study, we found that short‐term HFD feeding alone, without cisplatin, showed to cause a reduction of total glutathione (GSH, a most abundant antioxidant molecule) in the mitochondria. Also, cisplatin administration increased H_2_O_2_ formation in kidney tissues and decreased reduced form of GSH and MnSOD, and IDH2 expression in the mitochondrial fractions of kidney tissues and these changes were greatly augmented by HFD feeding. In addition, treatment of mitochondrial antioxidant during HFD feeding and fasting protect kidneys against cisplatin. Andrich et al. reported that total GSH levels decreased and oxidative stress increased in skeletal muscles of HFD fed rats for 2 weeks (Andrich et al., 2019), partially supporting our data. Furthermore, in this present study, Mito‐TEMPO demonstrated greater protection in HFD mice compared to LFD. This indicates that HFD intake for only for 2 weeks may increase the susceptibility of cells to oxidative stress, consequently increasing kidney susceptibility to cisplatin. If the duration of diet intake or amount of calorie intake were defined by further studies, the results will be more useful to the clinic.

Several studies have reported that cisplatin nephrotoxicity is accompanied by significant elevations of total cholesterol and triglyceride concentrations in blood, leading to lipid accumulation in kidney tissue (Jang et al., 2020; Li et al., 2012; Portilla et al., 2006). Lipolysis in adipose tissue, a major store of cholesterol, is critical for the regulation of circulating cholesterol levels (Krause & Hartman, 1984; Verghese et al., 2007) and increased lipolysis in adipose tissue causes lipotoxicity in the tissues by releasing lipids into the bloodstream and subsequently accumulating lipids into the peripheral tissues (Nishi et al., 2019). Li et al. reported that cisplatin increased lipid accumulation in the kidney proximal tubule cells, suggesting that it is associated with destruction and lipolysis by cisplatin‐mediated systemic inflammation in adipose tissue (Li et al., 2012). It has been known that an excessive accumulation of mitochondrial cholesterol affects mitochondrial function including oxidative phosphorylation (Solsona‐Vilarrasa et al., 2019). Furthermore, it has been reported that mitochondrial cholesterol loading impairs the transport of mitochondrial GSH in hepatic cells, resulting in depletion of mitochondrial GSH and increased susceptibility to oxidative stress (Baulies et al., 2018). Criscuolo et al. reported that cisplatin‐resistant ovarian cancer cells show reduced cholesterol levels and down‐regulation of cholesterol in those cells increases the resistance of cisplatin‐induced apoptosis (Criscuolo et al., 2020). Mei et al. reported that a high cholesterol diet induces renal apoptosis, inflammation, and oxidative stress (Mei et al., 2020). In this present study, we found that HFD feeding increased blood cholesterol and kidney lipid levels, and decreased mitochondrial total GSH levels. In addition, cisplatin‐induced increases in cholesterol levels in the blood and kidney tissue and the decreases of total GSH levels and the ratio of reduced GSH to tGSH in the mitochondria. And HFD‐feeding augmented cisplatin‐induced those mentioned above. Therefore, we speculate that increased blood cholesterol and lipid accumulation in the proximal tubular cells and decreased mitochondrial GSH due to HFD‐feeding augment the cisplatin‐mediated lipolysis activation lipid accumulation, and the reduction of GSH, leading to increased cisplatin‐induced mitochondrial oxidative stress and kidney damages. Supporting this, Elsayed et al. reported that cisplatin increased circulating cholesterol, and that antioxidant treatment inhibits the cisplatin‐induced increase of blood cholesterol concentration, along with reduced cisplatin‐induced kidney damage and oxidative stress (Elsayed et al., 2021). Likewise, in this current study, we also found that Mito‐TEMPO, a mitochondrial antioxidant, treatment reduced the increases of blood cholesterol concentration after cisplatin administration.

Under pathological and physiological conditions, the structure and function of mitochondria are delicately preserved by fission and fusion, which can induce shortening and elongation of mitochondria, respectively, and the disturbances of the mitochondrial dynamics can lead to mitochondrial dysfunction and consequently cellular damage. Recent studies have demonstrated that excess nutrient intake causes impairments of normal mitochondrial dynamics, leading to activation of apoptosis signaling pathway and that oxidative stress impairs mitochondrial dynamics (Cunarro et al., 2018; Liesa & Shirihai, 2013; Qiu & Schlegel, 2018; Yu et al., 2008). Sun et al. reported that long‐term high‐calorie intake increases the susceptibility of kidneys to cisplatin through increased oxidative stress and mitochondrial fission in mice (Sun et al., 2020). Yu et al. reported that high glucose treatment in cultured cells induces the impairment of mitochondrial dynamics by rapid mitochondrial fission with a concomitant increase in ROS production (Yu et al., 2008). In addition, it has been reported that mitochondrial elongation was associated with reduced mitochondrial ROS production, whereas mitochondrial fragmentation increases mitochondrial ROS production (Picard et al., 2013). In contrast, studies also have demonstrated that mitophagy preceded by mitochondrial fission, is beneficial for cells by mitochondrial quality control by removal of unhealthy mitochondria (Larson‐Casey et al., 2016; Ma et al., 2020). Kume et al. demonstrated that in the aged mouse kidney, long‐term calorie restriction adaption to hypoxia through Sirtuin 1 (SIRT1) dependent mitophagy (Kume et al., 2010). In this present study, we found that HFD‐feeding alone induces increased Fis1, conversely, decreased Opa1 expression, and that cisplatin increased mitochondrial fission protein expression, conversely, decreased opa1 expression, and HFD‐feeding augmented those cisplatin‐induced changes. In addition, treatment of Mito‐TEMPO during HFD feeding prevented cisplatin‐induced changes and fasting also prevented those. Therefore, we speculate that cisplatin impairs normal mitochondrial dynamics and HFD‐feeding potentiates this impairment, inducing mitochondrial damage and apoptosis. Supporting this, studies have indicated that obesity induces mitochondrial dysfunction and a shift of the mitochondrial dynamics towards fission, leading to mitochondrial fragmentation and activation of the apoptotic signal pathway (Archer, 2013; Brooks et al., 2009). Furthermore, it has been reported that increased cholesterol accumulation in mitochondria also is the cause of mitochondrial dysfunction (Montero et al., 2010; Solsona‐Vilarrasa et al., 2019; Torres et al., 2019). Solsona‐Vilarrasa et al. reported that an increase in mitochondrial cholesterol content increases the fluidity of the mitochondrial membrane (Solsona‐Vilarrasa et al., 2019). Montero et al. reported that cholesterol‐mediated mitochondrial GSH depletion stimulates cardiolipin, destabilizing the lipid bilayer and enhancing Bax‐induced mitochondrial membrane permeability (Montero et al., 2010). Like this, increased mitochondrial cholesterol levels are associated with increased oxidative stress, impairment of mitochondrial dynamics, and susceptibility to apoptosis in various pathological conditions. These present studies show that increased susceptibility to cisplatin toxicity in the kidney after HFD intake is associated with increased mitochondrial damage by increased cholesterol level and oxidative stress, suggesting that calorie intake affects kidney susceptibility to injury. However, clinical application requires careful consideration of the fact that the patient may already be experiencing cachexia.

## 5 CONCLUSION

Our study demonstrates that short‐term high‐fat diet intake aggravates cisplatin‐induced kidney injury via increased oxidative stress of mitochondria. In contrast, short‐term fasting protects kidneys against cisplatin. This indicates that the food intake control, even for a short period, may be a strategy for the reduction of cisplatin‐induced AKI. Finally, our data clearly demonstrate that food supply control affects kidney susceptibility to cisplatin.

## CONFLICT OF INTERESTS

There are no conflicts of interest to declare.
